# Binderless Thermal Insulation Boards from Rapeseed Straw: Optimization and Performance Analysis

**DOI:** 10.3390/ma18245481

**Published:** 2025-12-05

**Authors:** Miloš Jerman, Martin Böhm, Jakub Vrzáň, Jitka Krejsová, Klára Kobetičová, Robert Černý

**Affiliations:** Department of Materials Engineering and Chemistry, Faculty of Civil Engineering, Czech Technical University in Prague, Thákurova 7, 166 29 Prague, Czech Republic; martin.bohm@fsv.cvut.cz (M.B.); jakub.vrzan@fsv.cvut.cz (J.V.); jitka.krejsova@fsv.cvut.cz (J.K.); klara.kobeticova@fsv.cvut.cz (K.K.); cernyr@fsv.cvut.cz (R.Č.)

**Keywords:** rapeseed straw, binderless insulation boards, thermal conductivity, water vapor diffusion, hygrothermal performance, lignin self-bonding

## Abstract

The development of sustainable thermal insulation materials is crucial for reducing the environmental impact of the construction sector. This study investigates the potential of binderless insulation boards made from rapeseed fibers, utilizing the natural adhesive properties of lignin. The effects of fiber fineness and processing temperature (160 °C and 180 °C) on basic physical, hygric and thermal properties were examined. The influence of temperature on thermal conductivity was minimal, while higher temperature slightly reduced moisture content and swelling. Finer fibers and higher temperature increased the water vapor diffusion resistance factor. Microscopy and thermal analyses confirmed sufficient lignin softening and fiber bonding at 160 °C, whereas higher temperatures caused partial fiber degradation. Overall, the results demonstrate that rapeseed straw boards provide a sustainable and vapor-permeable alternative for roof and general insulation applications, with processing conditions and fiber fineness influencing hygric properties more than thermal performance.

## 1. Introduction

The construction sector plays a major role in global energy consumption and greenhouse gas emissions, with heating and cooling of buildings representing a significant share of this demand [1,2]. One of the most effective strategies for reducing energy loss in buildings is the use of external thermal insulation composite systems (ETICS). However, conventional thermal insulation materials used in ETICS are typically produced from petrochemical sources, requiring high processing energy and exhibiting a high embodied carbon footprint [3,4,5,6,7]. These environmental concerns have accelerated the search for sustainable, bio-based alternatives with lower environmental impact.

Bio-based materials offer a substantially lower ecological footprint and may contribute to the decarbonization of the building sector [4,8]. While wood remains the primary lignocellulosic resource, growing concerns over forest depletion have increased attention toward non-wood lignocellulosic fibers for insulation applications [9,10,11].

Several natural materials have been investigated as alternatives to conventional insulation products due to their low embodied energy and regional availability. These bio-based sources can be categorized into non-woody agricultural residues and region-specific plant materials. Among region-specific resources, *Posidonia oceanica*, a Mediterranean seagrass, accumulates naturally on shorelines contains approximately 45% cellulose, 19.7% hemicellulose, and 31.5% lignin [12], which facilitates thermal self-bonding. Dried Posidonia fibers can be formed into boards with thermal conductivity values ranging from 0.036 to 0.045 W/(m·K) and bulk densities between 123 and 250 kg/m^3^, depending on binder content and processing conditions [13,14]. In addition, Posidonia has been used in composite materials [15] and even as blown insulation, with thermal conductivity ranging from 0.047 to 0.070 W/(m·K) [16]. Another promising tropical resource is Totora (*Schoenoplectus californicus*), a wetland plant native to the Americas, for which boards made with natural adhesives such as sodium alginate—or relying solely on lignin activation—achieved thermal conductivity values of 0.046–0.058 W/(m·K) [11,17]. Other tropical plants, including fast-growing palms [18], oil palm wood [19], kapok (*Ceiba pentandra*) [20], and jute (*Corchorus*) [6,21,22], have also been investigated. However, despite their favorable insulating properties, these materials are generally less suitable for inland European regions due to transportation-related emissions [23].

Europe offers large volumes of underutilized lignocellulosic residues, such as wheat and barley straw, hemp [24,25,26], sunflower bark [27], corn cobs [28,29,30], and rapeseed straw. Corn cobs can be milled and bonded with urea-formaldehyde resin to produce insulation boards [31]. Sunflower stalks have been incorporated into gypsum-fiber composites with moderate strength but acceptable thermal performance [32]. Other studies have developed fiberboards from sunflower using synthetic binders such as methylene diphenyl diisocyanate (MDI) and urea-formaldehyde resins, complying with standards such as EN 312 [23,33]. Nevertheless, the environmental burden and limited recyclability of such binders remain significant drawbacks [29,34].

Lignocellulosic fibers, composed of cellulose, hemicellulose, and lignin, are promising for binderless board production due to the thermoplastic behavior of lignin. When heated between 100 °C and 200 °C, lignin softens and migrates to fiber surfaces, forming localized inter-fiber bonds [35]. This self-bonding mechanism eliminates the need for synthetic adhesives and improves recyclability. Fiber morphology plays a critical role: finer fibers provide greater specific surface area and expose more hydroxyl groups, enhancing adhesion via hydrogen bonding and mechanical interlocking.

Rapeseed (*Brassica napus*) is a widely cultivated annual crop in Europe, primarily grown for vegetable oil production. Its residual biomass—rapeseed straw—represents a readily available and largely underutilized by-product. In the European Union alone, several million tons of rapeseed straw are generated annually [36,37], making it a promising feedstock for large-scale material applications.

Chemically, rapeseed straw contains approximately 19–21% lignin and about 40% cellulose [38,39,40,41,42], which makes it suitable for binderless fiberboard production. Compared to wood-based materials, it offers key advantages: it is an agricultural residue requiring no forest harvesting, and it is typically dry upon collection, reducing the need for additional energy-intensive drying processes.

In contrast to other agricultural residues, rapeseed straw has a favorable fiber structure and composition for thermal activation of lignin. This, combined with its high availability and fast annual regeneration cycle, positions it as a sustainable and regionally appropriate raw material for eco-friendly insulation systems.

This study evaluates the potential of producing binderless thermal insulation boards from rapeseed straw by activating lignin through controlled thermal processing. The effects of fiber fineness and pressing temperature (160 °C and 180 °C) on basic physical, hygric and thermal properties are investigated.

The novelty of the work lies in the optimization of a fully binderless, vapor-permeable insulation system based on a temperate-zone agricultural residue. Unlike previous studies that focused on tropical or marine plants, this study provides a comprehensive dataset on rapeseed straw, including sorption isotherms, water vapor diffusion resistance factor, and moisture-dependent thermal conductivity, which are essential for hygrothermal performance modeling in building envelopes.

## 2. Materials and Methods

### 2.1. Fiber Preparation and Board Manufacturing

Rapeseed straw was harvested near Polepy in the Bohemian Central Highlands (Czech Republic) and processed at the Institute for Wood Technology (IHD, Dresden, Germany). The material underwent steam pulping at 160 °C and 9 bar in a water vapor saturated environment. Three fiber grades were produced by adjusting the grinding disk distance: 0.14 mm (fine), 0.35 mm (medium), and 0.50 mm (coarse).

After defibration, fibers were manually distributed into 250 mm × 250 mm molds and lightly compressed under an aluminum plate to ensure uniform pressure. The mats were steam-treated at either 160 °C or 180 °C for 30 min under 8 kPa, mechanically clamped, and subsequently dried at 60 °C to constant mass. The final board thickness was 25 mm.

### 2.2. Fiber Length Analysis

**Figure 1:**
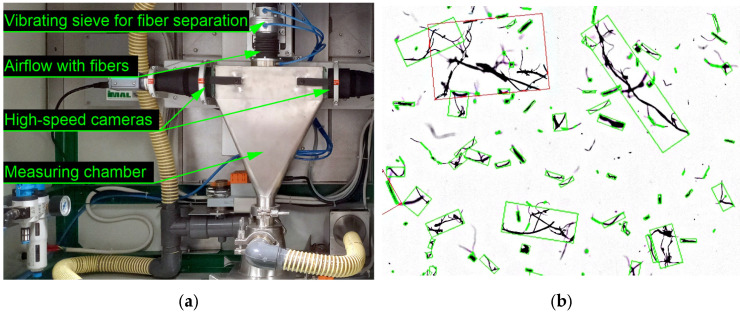
T Schematic view of the fiber length analysis system IMAL (**a**) and an example of image-based fiber detection (**b**). Only well-focused fiber projections (marked with green frames) were included in the analysis, while overlapping or clustered fibers (marked in red) were excluded.

The length distribution of rapeseed straw fibers was analyzed using the Fiber-Cam 100 system (IMAL, San Damaso, Italy), which utilizes high-speed imaging of particles suspended in an airflow (Figure 1). For each fiber grade (fine, medium, coarse), a representative 5 g sample was dispersed and measured in an airstream. Fiber length is one of several morphological parameters that influence bulk density, mechanical strength, and hygrothermal performance of lignocellulosic insulation materials; however, it does not fully define fiber fineness [37,38].

Previous studies have demonstrated that fiber size, typically expressed through length, thickness, or a combination thereof, can substantially affect panel performance. Ayrilmis et al. [43] reported that increasing fiber length in the core layer of MDF from 4.3 to 11.5 mm improved bending strength, bending modulus, and internal bond strength, with similar trends observed for thickness swelling until excessively long fibers reversed the effect. Likewise, Imken et al. [44] showed that longer softwood fibers tend to generate higher mechanical strength and lower thermal conductivity than shorter hardwood fibers, emphasizing the relevance of fiber-size distribution for insulation performance. Segovia et al. also confirmed the strong influence of fiber dimensions on mechanical properties of bio-based composites [45]. These findings demonstrate that fiber dimensions, rather than fineness in the narrow sense, represent a critical morphological factor influencing both mechanical and thermal behavior.

In this context, fiber length analysis was used to describe the morphological distinctions among the fine, medium, and coarse fractions generated by the fractionation process. This characterization enables a clearer interpretation of how differences in fiber-size distribution contribute to the physical and hygrothermal properties of the resulting insulation boards.

### 2.3. Thermal Analysis

Thermal behavior of rapeseed straw and processed fibers was investigated using simultaneous thermogravimetric analysis (TGA) and differential scanning calorimetry (DSC). The measurements were performed on a STA 449 F5 Jupiter thermal analyzer (NETZSCH, Selb, Germany) coupled with a quadrupole mass spectrometer. Alumina crucibles (Al_2_O_3_) were used for both the sample and reference pans.

Approximately 5 mg of material was analyzed in each test. The samples were heated from 35 °C to 800 °C under a dynamic argon atmosphere with a flow rate of 240.3 mL/min. The heating rate was set to 10 °C/min. Thermogravimetric data (mass loss and DTG) were recorded simultaneously with the DSC signal. Evaluation of thermal transitions and degradation stages was conducted using NETZSCH Proteus Thermal Analysis software, version 8.0.3.

The thermal analysis allowed identification of hemicellulose and cellulose degradation ranges, as well as the broad softening and decomposition interval characteristic of lignin. The comparison between raw straw and thermally processed fibers enabled qualitative assessment of component distribution and thermal stability after fiber manufacturing.

Only well-focused fiber projections (marked with green frames) were included in the analysis, while overlapping or clustered fibers (marked in red) were excluded. In each measurement, corresponding to approximately 5 g of fibers, between 59,000 and 81,000 individual fibers were analyzed (Figure 1).

### 2.4. Microstructure

The preliminary visual assessment of the fiber morphology was performed using a digital microscope camera Dino-Lite Edge (model AF4135ZTLE, AnMo Electronics Corporation, Taiwan) with DinoCapture 2.0 software. This method provided rapid, low-magnification documentation of fiber structure and surface characteristics under ambient conditions. Image labeling, scale calibration, and selected image analysis tasks were carried out using NIS-Elements BR software, version 5 (Laboratory Imaging, Prague, Czech Republic) (Nikon, Tokyo, Japan).

The microstructure of the insulation boards, including localized bonding zones formed between adjacent fibers, was analyzed using a desktop SEM Phenom XL (ThermoFisher Scientific, Waltham, MA, USA) equipped with a secondary electron detector. The microscope was operated at an accelerating voltage of 15 kV and a working distance between 5 and 15 mm. Samples were dried under vacuum at room temperature, mounted on aluminum stubs using conductive carbon tape, and sputter-coated with a approximately 5 nm layer of gold-palladium (80/20) using a Quorum SC7620 coater (Quorum Technologies Ltd., Lewes, UK).

For high-resolution imaging of individual fiber surfaces, an ultra-high resolution scanning electron microscope Tescan Amber X (Tescan Orsay Holding, Brno, Czech Republic) was used. This system enabled detailed surface analysis at magnifications exceeding 50,000×, allowing the assessment of surface features and potential thermal damage caused by processing. The instrument was operated under low-vacuum mode using secondary electron detector.

### 2.5. Basis Physical Properties

Bulk density was determined according to EN 1602 on five samples per board type (250 mm × 250 mm × 25 mm) by dividing dry mass by specimen volume [46].

Thickness swelling (TS) was assessed by immersing 100 mm × 100 mm samples in water at 20 °C for 24 h. For each board type, three samples were tested. Thickness was measured before and after immersion at four points per sample using a 0.05 mm dial gauge. TS was expressed as the relative increase in thickness. Water absorption (WA) was measured according to EN 317:1995 [47]. Dry sample mass was recorded, followed by 24 h immersion. WA was calculated as the percentage increase in mass.

### 2.6. Water Vapor Diffusion Properties

Water vapor diffusion properties were determined using the standard cup method without a temperature gradient. For each board type, three samples with a square cross-section with an edge length of 100 mm were tested. The samples were sealed over cups containing a solution of known relative humidity and placed in a controlled environment.

Both dry cup and wet cup variants were applied. Mass change was monitored periodically, and the water vapor flux was calculated from the linear portion of the mass vs. time curve. The water vapor diffusion coefficient (D) and water vapor diffusion resistance factor (μ) were derived from established equations. The measurement of dry cup and wet cup was performed according to the EN ISO 12572 [48] and it is detailed described, e.g., in Ref [49].

### 2.7. Sorption Isotherms

Moisture sorption behavior of the insulation boards was assessed using the gravimetric method under controlled climatic conditions. For each board type, five samples with dimensions of 20 mm × 20 mm × 20 mm were tested. The samples were first oven-dried under vacuum until reaching a constant mass. They were then placed in a climatic chamber (23 °C) at sequentially increasing relative humidity (RH) levels: 20%, 40%, 60%, 80%, 90%, and 98%.

At each RH step, the samples were conditioned until equilibrium was reached, defined as a mass change of less than 0.1% over 24 h. After stabilization, the sample mass was recorded using an analytical balance with 0.1 mg precision. The gravimetric moisture content (mass of absorbed water relative to dry mass) was then calculated for each humidity level.

### 2.8. Thermal Properties

Thermal conductivity as a function of moisture content and specific heat capacity were measured using a transient method with an ISOMET 2104 device (Applied Precision, Bratislava, Slovakia). For each board type, three samples were tested. The measurement involves evaluating the temperature response of the material to heat flow pulses. The heat flow is generated by an electrical resistor heater, which is in direct thermal contact with the sample. The measurements were conducted at a laboratory temperature of 22 °C, with variations in moisture content.

## 3. Results and Discussion

### 3.1. Fiber Length Analysis

**Figure 2:**
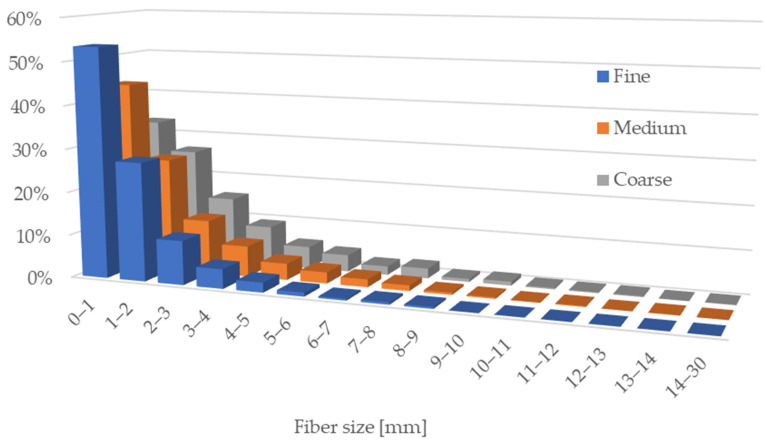
Fiber length distribution of rapeseed fibers in the fine, medium, and coarse groups.

The fiber length distributions (Figure 2) were classified into 15 intervals, each covering a 1 mm range from 0 mm up to 14 mm. An additional final interval included all fibers ranging from 14.01 mm to 30 mm. This classification enabled a detailed comparison of fiber size distribution across the fine, medium, and coarse fiber groups. From each 5 g fiber sample, the system recorded a total of 57,363 fibers in the fine group, 71,466 in the medium group, and 59,925 in the coarse group.

The highest proportion of fine particles was observed in the fine fiber group, produced using a disk spacing of 0.19 mm. This group contained the shortest fibers, with 53.4% having lengths between 0.1 and 1 mm. In comparison, the medium group showed approximately 10% fewer fibers in this range, while the coarse group had about 20% fewer. In the 1–2 mm interval, the fiber content was relatively similar across all groups, with the fine group having the highest value, although the difference did not exceed 1% compared to the others. From the 2–3 mm range onward, the distribution pattern gradually shifted. Longer fibers were most common in the coarse group, while the fine group exhibited no presence of fibers beyond the 9–10 mm range. In contrast, the medium group contained only trace amounts of long fibers in this range, typically below one percent. The coarse group also included the longest fibers (up to 30 mm), with the 14–30 mm interval accounting for 0.1% of the total.

These variations in fiber morphology affected the internal structure of the boards. Fine fibers formed a more compact and homogeneous matrix but provided weaker inter-fiber bonding due to limited mechanical entanglement. Coarse fibers enabled stronger physical interlocking but resulted in more voids and heterogeneity. The medium group offered a compromise between density and cohesion, suggesting a favorable balance for mechanical stability and processing efficiency.

### 3.2. Thermal Analysis

**Figure 3:**
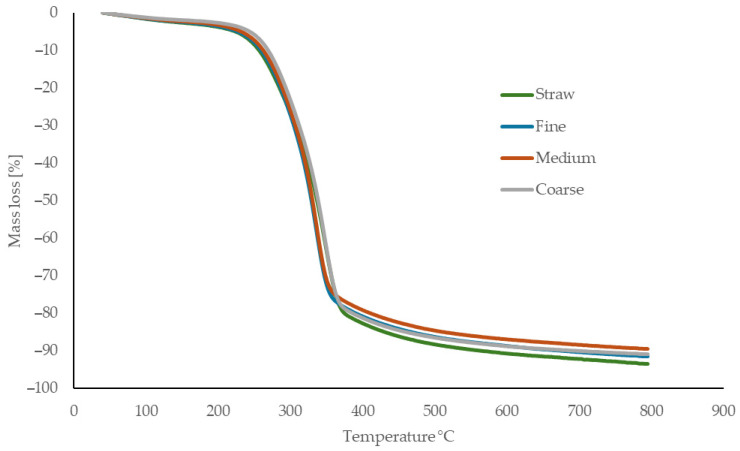
Thermogravimetric curves of untreated straw and processed rapeseed fibers (fine, medium, coarse).

**Figure 4:**
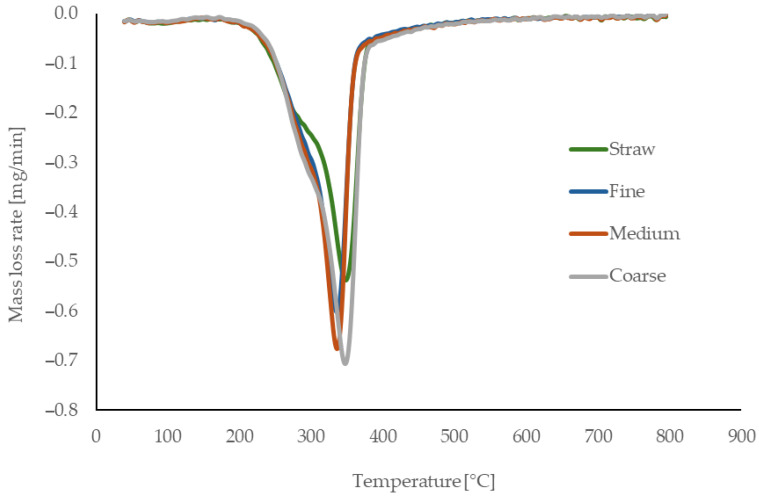
Derivative thermogravimetric curves of untreated straw and manufactured rapeseed fibers (fine, medium, coarse).

**Figure 5:**
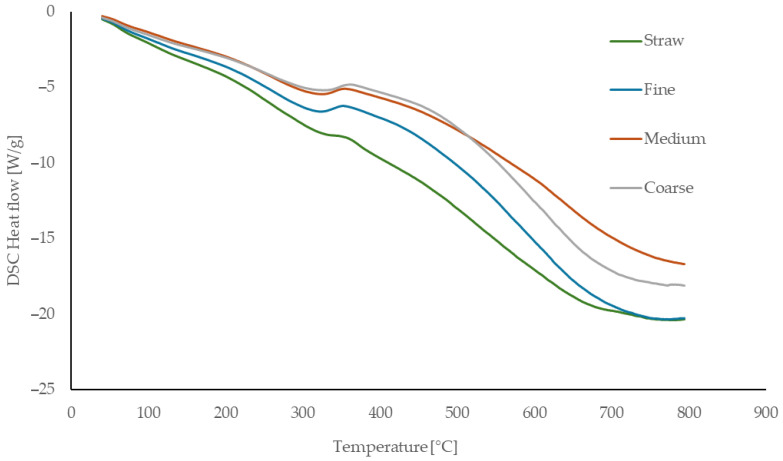
DSC curves of untreated straw and manufactured rapeseed fibers (fine, medium, coarse).

The thermal behavior of the reference material (untreated rapeseed straw) and the processed fibers used for binderless board production is presented in Figure 3 (TG), Figure 4 (DTG), and Figure 5 (DSC). The reference rapeseed straw was not subjected to any thermal treatment prior to board manufacturing. In contrast, the fibers experienced elevated temperatures of up to 160 °C during mechanical defibration, which affected their thermal behavior.

Hemicellulose decomposition begins around 200 °C, with untreated straw showing a more pronounced mass loss due to its higher hemicellulose content, while processed fibers demonstrate lower activity, likely reflecting partial thermal degradation during processing. Cellulose decomposition initiates near 255 °C, with a sharp increase in the degradation rate up to approximately 300 °C, consistent across all samples. The initial phase is endothermic, driven by moisture loss and structural transitions, followed by an exothermic peak between 331 and 350 °C corresponding to cellulose degradation. Lignin degradation progresses slowly above 400 °C, forming char and ash.

Finer fibers show slightly more pronounced exothermic peaks at high temperatures, likely due to increased surface lignin concentration resulting from mechanical defibration. Overall, thermal processing reduces low-temperature reactivity and enhances structural stability through partial component degradation and reorganization.

The thermal behavior of the fibers is consistent with literature data. Kačíková et al. [50] reported that moderate thermal exposure reduces hemicellulose-related mass loss, corresponding to the lower reactivity of processed fibers. Gezer and Kuştaş [51] showed that structural modification of lignocellulosic materials shifts the onset and intensity of decomposition, in agreement with the initial degradation observed in our samples. The main cellulose decomposition interval (255–300 °C) matches values reported by Rantuch and Chrebet [52]. Reference rapeseed straw analyzed by Zhang et al. [53] exhibited similar DSC and TGA transition temperatures, confirming the typical thermal decomposition pattern of rapeseed-based lignocellulosics.

### 3.3. Microstructure

**Figure 6:**
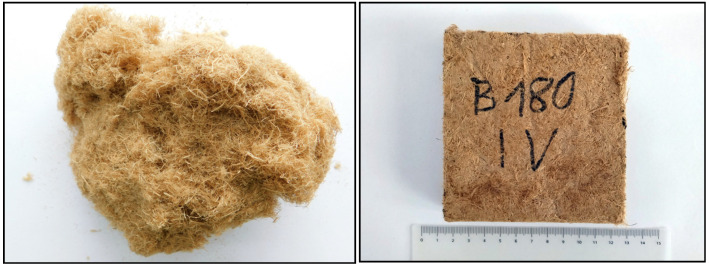
Macroscopic image of the produced fibers (**left**) and a representative sample manufactured at a pressing temperature of 180 °C for experimental testing (**right**).

From a macroscopic perspective (Figure 6), all fiber batches and the resulting insulation boards appeared visually similar, without marked surface differences.

**Figure 7:**
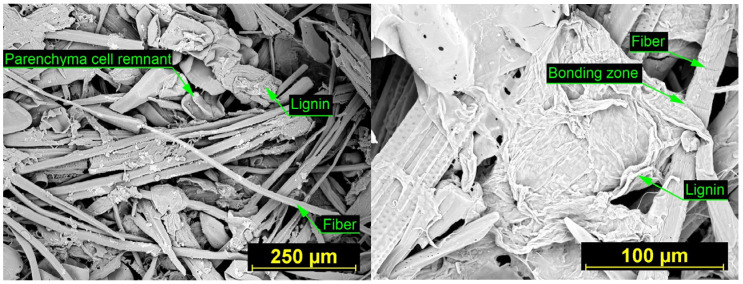
SEM images of the fiber surfaces in the insulation board produced at 160 °C. (**Left**): overview of the fiber network at 300× magnification. (**Right**): detailed view of the insulation board surface at 1000× magnification.

**Figure 8:**
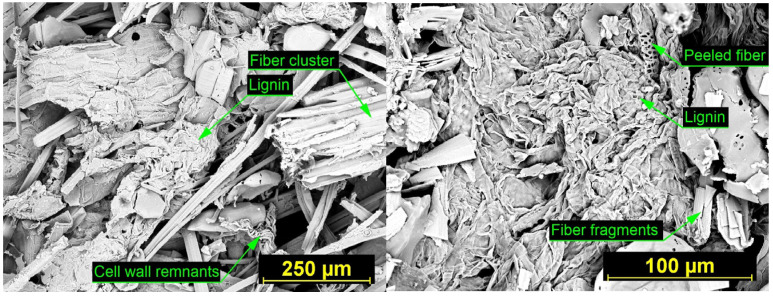
SEM images showing the microstructure of the insulation board produced at 180 °C. (**Left**): general view of the fiber arrangement at 300× magnification. (**Right**): close-up of the surface morphology at 1000× magnification, highlighting lignin distribution.

SEM images of insulation boards produced at 160 °C (Figure 7) and 180 °C (Figure 8) illustrate the effect of processing temperature on fiber morphology and inter-fiber bonding. At 160 °C, the board shows a relatively intact network of fiber bundles with limited signs of thermal degradation. Inter-fiber bonding zones are present but less pronounced, and the lignin remains largely confined within the fiber walls. In contrast, the board produced at 180 °C exhibits a more fragmented fiber structure, with numerous cell wall remnants and clearly visible spiral wall thickenings, indicating collapsed xylem elements. At this higher processing temperature, more extensive lignin softening and enhanced fiber-to-fiber contact were observed, contributing to improved structural integration of the material through the formation of well-developed lignin-rich bonding zones.

Both boards contain fiber debris, parenchyma cell remnants, and void spaces. However, the board produced at 180 °C shows a higher concentration of fragmented fiber material due to increased thermal degradation. At the same time, the more pronounced softening of lignin at this temperature promotes the formation of stronger and more continuous inter-fiber bonds, which further enhances the structural integrity of the insulation material.

### 3.4. Basic Physical Properties

The basic physical properties of the manufactured boards, including bulk density, water absorption, and thickness swelling, are summarized in Table 1.

As shown in Table 1, the bulk density of all boards ranged between 133 and 139 kg/m^3^, with slightly higher values for fine fibers. Water absorption was consistent across all groups (~6.17 kg/kg), and thickness swelling remained low, generally around 3–3.5%.

Boards pressed at 180 °C exhibited marginally reduced thickness swelling, indicating improved dimensional stability. Compared to earlier studies using coarser fibers, the current results show better swelling resistance, likely due to increased fiber compaction.

### 3.5. Water Vapor Diffusion Properties

The results obtained from both the dry cup and wet cup methods are summarized in Table 2. Samples produced from fine fibers exhibited a slightly higher water vapor resistance factor (μ). However, the differences between the individual types of manufactured boards are minimal and may be considered negligible in practical applications.

As shown in Table 2, only minor differences were observed among the tested boards. Dry cup μ-values ranged from 5.4 to 6.8, while wet cup values were lower (1.9–2.3), reflecting the contribution of capillary-condensed moisture transport in the wet cup method [54].

In comparison, *Posidonia oceanica* fiberboards reached substantially higher resistance (μ > 15.9, dry cup) [13]. Other vapor-permeable materials, such as Sphagnum moss, peat-moss mixtures, wood shavings, or paper wool, exhibited μ-values between 2.3 and 3.9 at densities of 40–90 kg/m^3^ [55], while jute, flax, and hemp products typically fall within the 2–4 range [49].

A clear trend was observed: higher bulk density tends to increase water vapor transport resistance. For example, rapeseed straw boards bonded with bone glue at 655 kg/m^3^ reached μ = 35 (dry cup) and 24 (wet cup) [36].The tested binderless boards (μ = 2.7–5.3) are fully vapor-permeable and meet the expected performance.

### 3.6. Sorption Isotherms

**Figure 9:**
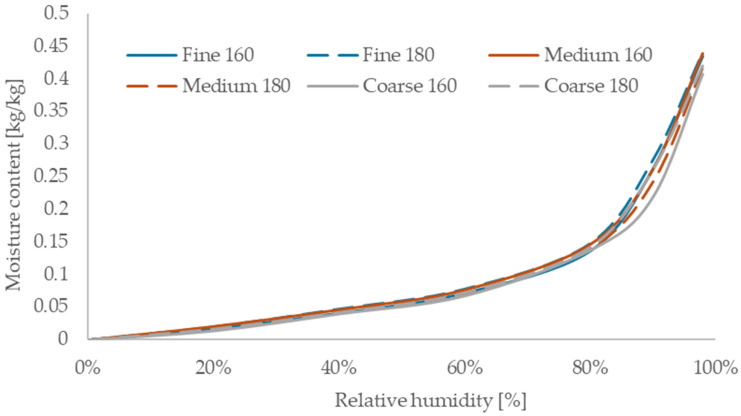
Sorption isotherms of the manufactured boards.

The moisture sorption isotherms of the tested boards are shown in Figure 9. All samples followed similar trends, but with distinguishable differences in magnitude. The boards made from fine fibers exhibited the highest sorption across the entire relative humidity range. Among them, the sample processed at 180 °C showed the highest total moisture adsorption, while the same fiber grade treated at 160 °C had slightly lower values. In contrast, the coarse-fiber board produced at 180 °C showed the lowest sorption capacity.

These results indicate that fiber fineness has a stronger effect on moisture adsorption than pressing temperature. Finer fibers provide greater specific surface area and expose more hydroxyl groups, which enhances water adsorption through hydrogen bonding.

The observed sorption behavior is comparable to other vapor-permeable bio-based insulations. For instance, jute boards showed similar sorption isotherms [48], though slightly lower than those observed in this study. Previous research on rapeseed straw [34] reported lower moisture adsorption, likely due to differences in fiber morphology or board density.

Overall, the sorption characteristics of the boards are consistent with those of cellulose-based insulation materials, as reported by Hurtado et al. [4], and confirm their suitability for buffering indoor humidity.

### 3.7. Thermal Properties

**Figure 10:**
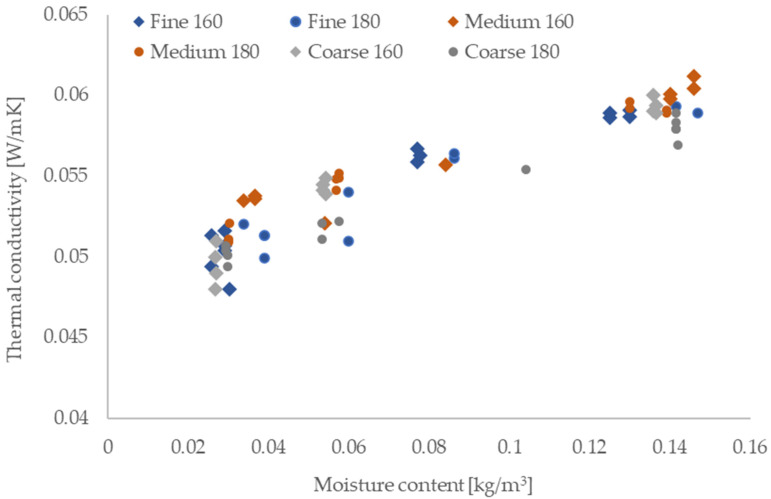
Thermal conductivity of the manufactured boards as a function of moisture content.

The measured thermal conductivity of the manufactured boards ranged from 0.0513 to 0.0533 W/(m·K), with little variation across fiber types and temperatures (Table 3). A clear linear relationship was observed between moisture content and thermal conductivity (Figure 10), which was consistent with the hygroscopic behavior of lignocellulosic materials.

These values are typical for bio-based insulations with bulk densities above 130 kg/m^3^. For comparison, *Posidonia oceanica* boards with similar densities achieved λ-values between 0.036 and 0.045 W/(m·K) [13], while Totora-based boards had thermal conductivity from 0.046 to 0.058 W/(m·K) depending on processing conditions [9]. Rapeseed boards thus fall on the higher end of the range but remain within the expected spectrum for plant-based insulations such as wood wool, hemp, flax, or sheep wool [49].

Lower thermal conductivity values in some cases, such as loose-fill Posidonia insulation (0.039 W/(m·K) at 60 kg/m^3^), illustrate the importance of optimizing bulk density. At too low densities, increased radiative transfer leads to higher overall λ-values [56]. This trade-off between conduction and radiation must be considered in design.

The specific heat capacity (cp) ranged between 1410 and 1457 J/(kg·K), which exceeds typical values for mineral wool and polystyrene, and is comparable to other bio-based materials. Although somewhat lower than the values reported for Posidonia [16], the measured cp still contributes to increased thermal inertia and improved indoor temperature buffering.

## 4. Conclusions

Binderless insulation boards from rapeseed straw were successfully fabricated by activating lignin through controlled thermal processing. The main findings are summarized as follows:

- Optimal processing temperature: A pressing temperature of 160 °C was sufficient to soften lignin and enable strong fiber bonding without damaging cellulose. At 180 °C, partial fiber degradation occurred.
- Effect of fiber fineness: Boards made from finer fibers and processed at 160 °C exhibited lower bulk density, more uniform lignin distribution, and enhanced vapor permeability, indicating a strong influence of fiber morphology on both physical and hygric behavior.
- Density and thermal performance: The produced boards had bulk densities about 50% lower than conventional softwood fiberboards while maintaining comparable thermal conductivity.
- Hygrothermal behavior: The vapor diffusion resistance factor (2.7–5.3) confirmed a vapor-permeable structure, similar to softwood fiberboards and higher than flax-, hemp-, or cellulose-based insulations.
- Application potential: Binderless rapeseed fiberboards represent a promising bio-based alternative for construction applications requiring moderate insulation performance, vapor permeability, and environmental sustainability, such as roof sheathing, exterior walls, or underfloor insulation.

## Figures and Tables

**Table 1:** Basic physical properties of the manufactured boards.

	Bulk Density [kg/m^3^]	WA 24 h [kg/kg]	TS 24 h [%]
Fine—160 °C	138 ± 8	6.17 ± 0.18	3.5 ± 0.11
Fine—180 °C	139 ± 8	6.18 ± 0.14	3.3 ± 0.10
Medium—160 °C	135 ± 5	6.16 ± 0.17	3.4 ± 0.12
Medium—180 °C	137 ± 6	6.17 ± 0.12	3.1 ± 0.10
Coarse—160 °C	133 ± 7	6.16 ± 0.12	3.4 ± 0.16
Coarse—180 °C	135 ± 8	6.18± 0.11	3.2 ± 0.15

**Table 2:** Water vapor diffusion properties of fiberboards.

Material	Water Vapor Diffusion Coefficient [×10^−6^ m^2^s^−1^]		Water Vapor Diffusion Resistance Factor [-]	
	Dry Cup	Wet Cup	Dry Cup	Wet Cup
Fine—160 °C	3.81 ± 0.36	10.4 ± 1.35	6.08 ± 0.57	2.21 ± 0.11
Fine—180 °C	3.48 ± 0.57	12.4 ± 2.05	6.81 ± 1.30	1.94 ± 0.38
Medium—160 °C	3.89 ± 0.32	10.1 ± 1.32	5.94 ± 0.51	2.29 ± 0.13
Medium—180 °C	3.82 ± 0.10	12.5 ± 2.64	6.04 ± 0.30	1.94 ± 0.53
Coarse—160 °C	4.28 ± 0.31	10.6 ±1.45	5.38 ± 0.30	2.17 ± 0.17
Coarse—180 °C	3.94 ± 0.18	10.5 ± 1.42	5.73 ± 0.47	2.19 ± 0.52

**Table 3:** Water vapor diffusion properties of fiberboards.

	Thermal Conductivity [W/m·K]	Specific Heat Capacity [J/(kg·K)]
Fine—160 °C	0.0521 ± 0.004	1410 ± 150
Fine—180 °C	0.0533 ± 0.003	1442 ± 171
Medium—160 °C	0.0517 ± 0.008	1443 ± 182
Medium—180 °C	0.0519 ± 0.002	1417 ± 154
Coarse—160 °C	0.0513 ± 0.002	1445 ± 168
Coarse—180 °C	0.0521 ± 0.002	1457 ± 126

## Data Availability

The original contributions presented in the study are included in the article/Supplementary Material, further inquiries can be directed to the corresponding author.

## Supplementary Materials

The following supporting information can be downloaded at: https://www.mdpi.com/article/10.3390/ma18245481/s1.
